# Rehydration Post-orientation: Investigating Field-Induced Structural Changes via Computational Rehydration

**DOI:** 10.1007/s10930-023-10110-y

**Published:** 2023-04-08

**Authors:** Maxim N. Brodmerkel, Emiliano De Santis, Carl Caleman, Erik G. Marklund

**Affiliations:** 1 Department of Chemistry – BMC, Uppsala University, Box 576, 75123 Uppsala, Sweden; 2 Department of Physics and Astronomy, Uppsala University, 75120 Uppsala, Sweden; 3 Center for Free-Electron Laser Science, DESY, Notkestrasse 85, 22607 Hamburg, Germany

**Keywords:** Molecular dynamics simulation, Protein hydration, Electric dipole, Protein structure, Structural biology, X-rays

## Abstract

Proteins can be oriented in the gas phase using strong electric fields, which brings advantages for structure determination using X-ray free electron lasers. Both the vacuum conditions and the electric-field exposure risk damaging the protein structures. Here, we employ molecular dynamics simulations to rehydrate and relax vacuum and electric-field exposed proteins in aqueous solution, which simulates a refinement of structure models derived from oriented gas-phase proteins. We find that the impact of the strong electric fields on the protein structures is of minor importance after rehydration, compared to that of vacuum exposure and ionization in electrospraying. The structures did not fully relax back to their native structure in solution on the simulated timescales of 200 ns, but they recover several features, including native-like intra-protein contacts, which suggests that the structures remain in a state from which the fully native structure is accessible. Our findings imply that the electric fields used in native mass spectrometry are well below a destructive level, and suggest that structures inferred from X-ray diffraction from gas-phase proteins are relevant for solution and in vivo conditions, at least after in silico rehydration.

**Supplementary Information:**

The online version contains supplementary material available at 10.1007/s10930-023-10110-y.

## Introduction

Proteins are biomolecules that underpin the workings of life as we know it today. Understanding their roles and functions naturally constitutes an immensely important task for scientists, and is closely linked to understanding their structures. Whilst the native environment for proteins is most often aqueous, structural biology and related research areas frequently employ techniques and methods that probe structures under other or after vacuum-exposure.

**Fig. 1:**
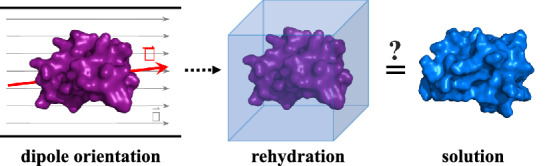
Rehydrating dipole-oriented proteins. Applying an EF $\vec {E}$ allows to influence the dipole $\vec {\mu }$ of a protein, orienting it along the EF direction [15]. However, dipole-orientation could induce permanent effects on the protein structure, promoting the rehydration of the thus obtained structures to refine the gained information even further. By comparing the post-orientation rehydrated structures with representative solution data, one can investigate occurring structural dissimilarities, and how they potentially revert back to the norm

X-ray crystallography [1] and various electron microscopy techniques [2, 3] long have been established for determining or probing protein structures. Such methods are immensely powerful and have provided vast numbers of high resolution structures, but also come with limitations. Despite recent advances, the experimental requirements (solubility, concentrations, etc.) and timescales complicates or effectively precludes the study of many protein systems, especially in rare and transient but biologically important states. Mass spectrometry (MS) is an alternative technique that has grown in popularity over the recent years, following a rapid development of technology and related methods. MS involves a separation and quantification of the molecules in solution after aerosolizing and ionizing them using electrospray ionization. This enables it to be applied to highly heterogeneous samples, and the wide range of MS variants provides structural information ranging from microns to Ångströms [4]. In native MS the experimental conditions are such that large intact non-covalently bound biomolecular complexes can be analyzed, which has transformed its utility for structural biology [5–9]. Nevertheless, despite high mass resolution, the structural resolution of native MS is limited [10]. Single particle imaging (SPI) experiments on the other hand have promised to enable high-resolution structure determination of single, non-crystalline samples in the gas phase through irradiation with short X-ray free-electron laser pulses [11]. Different injection methods have been proposed and tested, where native MS is a particularly attractive option, in part because of its non-destructive nature, separating ability, and the wide range of manipulations it offers [12]. During the process, the imaged particles are ultimately destroyed due to the extreme radiation [11, 13]. However, if the X-ray pulse length is chosen correctly, photons are scattered just before a particle explodes, thus allowing the reconstruction of the structure from the diffracted photons from a sequence of irradiated particles [11, 14]. The amount of data needed to be collected for a single SPI experiment is immense as a large amount of information is required to obtain a meaningful and representative structural model. Partially responsible for this is the random, uncontrolled spatial orientation of the particles during X-ray pulse irradiation. Theoretical gas-phase molecular dynamics (MD) simulations, a computational method providing atomistic information about the dynamics of molecules, provided by Marklund et al., showed that the orientations of proteins can be influenced by applying an electric field (EF) [15]. Most molecules possess an electric dipole, which will align its orientation along the direction of the EF [16]. For SPI experiments, this would reduce the needed amounts and also quality of the data for structural reconstruction [15]. The applied EF strength must be chosen with care however, as high EF values were proven to orient a protein very quickly, but in a destructive manner, leading to protein unfolding [15, 17] (Fig. 1).

Also in the absence of strong electric fields, solvent-free or even vacuum conditions can affect protein structures. On long time scales [18] or under activating conditions [19, 20], the structural loss can be considerable, but on shorter timescales relevant to SPI and many MS applications, proteins are often kinetically trapped in native-like structures with little or moderate alteration to the overall structure [19, 21–24]. For SPI, the structural variations between the individual proteins might be a larger problem than the actual radiation damage that ultimately leads to destruction of the protein [25]. Structural changes upon dehydration pertain not only to SPI and MS however. For example, protein-based drugs [26] are stored in a dry solid phase, then transferred to solution either prior to being administered or when they dissolve in the body. Consequently, the knowledge about how de- and rehydration affect the protein structure is crucial for ensuring the effectiveness of the drug and thus of considerable commercial value [27].

In a recent study we have shown that the rewetting of vacuum-exposed proteins leads to a nearly complete recovery of their native solution structures, especially when considering their inter-residue contacts [28]. Moreover, our results suggest that differences between different protein conformations persist after time spent in vacuo and after rehydration. Here, rehydration MD simulations displayed the potential to reverse the vacuum-induced structural changes, demonstrating MD as an powerful means to refine structures acquired from gas-phase experiments [28, 29]. Because it is virtually agnostic with regards to experimental details but still adaptable to different conditions, such rehydration in silico using MD can be of great utility for different gas-phase techniques and methods for structural biology, increasing the useful information from the experiment and the quality of any inferred structure models.

From the different aspects of sample injection, orientation and imaging, one question arises: *Do proteins exposed to external EFs find their way back to similar solution conformations, or does the exposure induce permanent alterations to the structures?* In order to answer this, we employed the results of Marklund et al. [15] as input to a new investigation with thorough rewetting MD simulations of four proteins—tryptophan cage (Trp-cage), the C-terminal fragment (CTF) of the L7/L12 ribosomal protein, ubiquitin and lysozyme—which were dipole-orientated in two different EFs, which were compared to simulations of rewetted gas-phase proteins without EF exposure. Furthermore, comprehensive solution simulations were conducted in order to obtain equilibrated solution data of the proteins for additional comparison. As such, our investigation separates any EF effects on the structures from the effects of vacuum exposure, and benchmarks them with native solution dynamics. This adds a new dimension to both dipole orientation and in silico rehydration, and furthers the knowledge about the structures and dynamics of gas-phase proteins.

## Methods

Four proteins—Trp-cage, CTF, ubiquitin and lysozyme—were investigated in their capability of potentially recovering their native solution conformations after dipole-orientation in vacuo in order to determine if the respective EF strength induces permanent conformational changes. Data from Marklund et al. [15] was taken as input, five replica starting structures per protein, obtained from simulations with EF strengths 0.0 V/nm, 0.2 V/nm and 0.4 V/nm.

### Rehydration Simulations

All simulations were conducted on the *Rackham* cluster of the Uppsala Multidisciplinary Center for Advanced Computational Science (UPPMAX) supercomputer. The Gromacs simulation package of version 2019.1 [30] was employed for the computations, using the OPLS-AA force field [31] and virtual sites for hydrogens [32]. The proteins were placed in a simulation box of dodecahedron geometry and under periodic boundary conditions, and solvated with water of the TIP4P model [33]. The net charge of each protein was determined by the $pK_{a}$ of the amino acid side chains at neutral pH, and the saline concentration was adjusted to 154 mM by adding sodium and chlorine ions.

The steepest descent algorithm was used to minimise the energy within each simulation system, followed by a short 50 ps MD simulation with applied position restrains. Subsequently, the temperature was adjusted to 300 K using the velocity rescaling thermostat [34] over 4 ns at a coupling constant of $\tau$ equal to 0.2 ps, where all bonds were constrained by the LINCS algorithm [35]. The pressure was modulated by employing the Berendsen barostat [36] with a coupling constant of $\tau$ equal to 0.1 ps, adjusting the simulation box volume to maintain a pressure value of 1 bar, over a 4 ns simulation as well with LINCS-constrained bonds. Afterwards, the dynamics of the proteins were captured at a time step of 4 fs over a duration of 200 ns in an isobaric, isothermal ensemble. Electrostatic interactions for all here presented simulations were computed utilising the particle mesh Ewald algorithm [37] at a real-space cut-off of 1 nm.

### Control Solution Simulations

Investigating the potential recovery of the native solution structure for a protein after vacuum exposure requires a data set to compare to as control. Therefore, solution MD simulations were conducted for each protein, however starting from structures obtained from their respective protein data bank entries: 1L2Y (Trp-cage), 1CTF (CTF), 1UBQ (ubiquitin), 1AKI (lysozyme). The simulation protocol to obtain solution simulation data as control was kept exactly the same as shown for the rehydration simulations, with the only differences being the starting structures being provided from the protein data bank. Moreover, after pressure coupling, a 100 ns long relaxation simulation was performed in order to provide five solvent-equilibrated structures for the production run.

### Root Mean Square Deviation and Fluctuation

All root mean square deviation (RMSD) calculations were performed with the Gromacs command rms [30]. Three different reference files were used for the RMSD computations: the first frame of each individual trajectory, the final structures at 200 ns from the control simulations, and the final structures at 200 ns from the rehydration simulations for 0.0 V/nm EF strength. Here, each trajectory was compared within their respective replica simulation to their ‘parent’ structure, allowing to follow their specific dynamics more independently. Afterwards, the individual RMSD trends for each calculation was averaged over all five replicas.

The root mean square fluctuation (RMSF) was calculated by concatenating all obtained trajectories belonging to the same set of replica simulations into a single trajectory. The average structure was calculated for the resulting trajectory, which was then used as reference structure to compute the residue-based RMSF of the concatenated trajectory. Moreover, to complement the RMSD and RMSF calculations, the number of hydrogen bonds between protein and solvent was calculated using the rms, rmsf, and hbond tools implemented in Gromacs [30].

### Interrogating Protein Dimensions

To further obtain valuable information about conformational changes for each protein during the MD simulations, we calculated the collision cross section (CCS), solvent-accessible surface area (SASA) and total protein volume. The theoretical CCS of the proteins was calculated using the Ion Mobility Projection Approximation Calculation Tool (IMPACT) [38] for the vacuum structures and structures belonging to the final 50 ns of the MD simulations, both for the control and rehydration. To complement the CCS results, we further computed the average SASA and total volume of the proteins, which were calculated with the respective Gromacs-supplied analysis tools [30]. The thus obtained data was averaged over the last 50 ns for all replicas, and the standard deviation calculated to estimate the differences between the individual values.

### Contact Maps

The MDAnalysis python package [39, 40] was employed for the generation of the contact maps. Initially, all atom-atom distances were calculated for the last 50 ns of the solution and rehydration simulation data. Contacts between two amino acids were defined as existing if the distance between at least one atom of each residue was equal to or smaller than 3.5 Å. Existing contacts were assigned a value of 1, and non-existing contacts a value of 0, which allowed us to identify the average occupancy over all simulation replicas for each residue-residue contact.

## Results

Several analyses were conducted to investigate the specific dynamics of the proteins and gather information to determine if EF orientation alters their structure significantly. MD simulation data analysis include RMSD and RMSF calculations, computing the CCS and the intramolecular contacts.

### Proteins Depart from Their Vacuum Structures Upon Rehydration

**Fig. 2:**
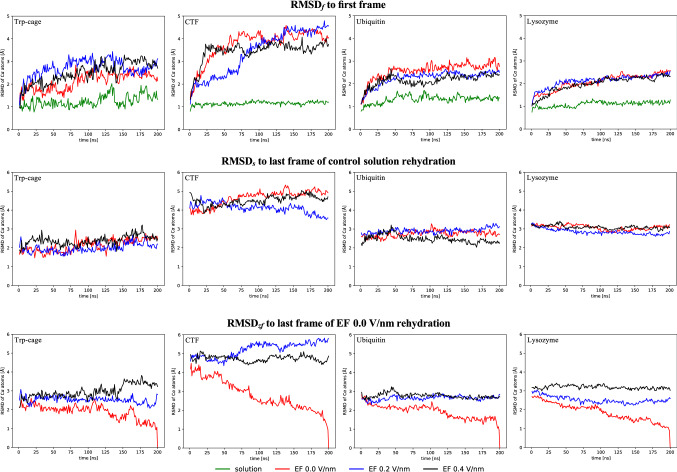
RMSD evolution over time. In the upper plots, the RMSD was calculated using to the initial structure of each trajectory as reference. The RMSD presented in the lower plots was calculated respective to the final structures of the control and zero-field rehydration simulation

Rewetting structures that were exposed to vacuum under different conditions, here under different EFs, provides information about similarities and dissimilarities between the structures. By calculating RMSD of the C$_\alpha$ atoms in the simulation trajectories relative to the appropriate reference structures, insights into the dynamics of rehydration might be obtained, allowing us to draw conclusions about structural effects of EF dipole orientation on proteins. We calculated RMSDs with three different reference structures: the initial structures of each trajectory (RMSD$_f$), to observe how the structures adapt to the solvent, the final structures of the control simulations in solution (RMSD$_s$), and the rehydration simulation originating from the zero-field (0.0 V/nm EF strength) gas-phase structures (RMSD$_{zf}$; see Fig. 2). Together, the different RMSDs gives information about the rehydration of each protein and how much the oriented proteins deviate from non-exposed gas-phase proteins.

For all proteins, the RMSD$_f$ for the control simulations were approximately 1 Å (Fig. 2), in line with accounts in literature [41–47]. The RMSD$_f$ for the rehydrated systems were notably higher, ranging from 2 to over 4 Å depending on the protein. CTF stands out with the highest RMSD$_f$. In an earlier study of the same proteins, CTF had high RMSDs compared to its solution structure [21], and in our original study on field orientation, CTF had the highest RMSD of all proteins without field exposure. Both facts point to the CTF structure being particularly affected by the vacuum conditions, which could explain why it also changes a lot when being rehydrated. We note that for CTF, the 0.2 V/nm variant had a still-increasing RMSF$_f$. This could be due to a still changing structure, but could also just be that its RMSD increase happened at a later point than the other variants.

The structures clearly change when reintroduced to bulk water. To get a first indication if the structures revert back to their native structures, we plotted the RMSD$_s$ for all rehydrated proteins. Here, constant low values would mean that the rehydrated structures were already close to their fully native counterparts, decreasing values would signify that they approach their native states, whereas stable high or increasing values would indicate that the rehydrated structures remain distinct from the native structures, or even diverge from them further over time. The RMSD$_s$ remained approximately constant for all proteins, at levels similar to their final RMSD$_f$. This indicated that the backbone were not able to recover from any distortions acquired during vacuum exposure over the rehydration simulation time scale. This resembles our earlier investigation of the bMS2 virus capsid dimer, where RMSDs remained high when compared to the solution structure [28]. Interestingly, prior field exposure made no striking difference for the RMSDs with respect to the first frame or to the control simulations, suggesting it had no impact on the overall structural difference brought on by rehydration. To explore the effect of the field exposure a bit more, we examined the RMSD$_{zf}$. By default, the zero-field simulations declined to zero values at the last time point (since that was the reference structure). All field-exposed proteins remained at approximately constant levels however, indicating that any differences in the backbone structures remain upon rehydration, and that they do not converge to a common structure in solution on these time scales. This could mean that the field causes specific structural changes that differ from the zero-field systems. However, the RMSD$_{zf}$ remained relatively high for the latter until a late time point, which rather suggests that vacuum exposed structures are less defined, that their backbones remain affected by the vacuum also after time spent in solution. This can be partly explained by an earlier observation that replica simulations of proteins in the gas phase starting from similar structures quickly show larger differences between each other than different time points within a single replica [48]. With that in mind, a likely interpretation is that the dehydration causes small backbone perturbations that differ between replicas, which remain for some time after initial rehydration.

### Dynamics During Rehydration is Insensitive to Prior Field Exposure

**Fig. 3:**
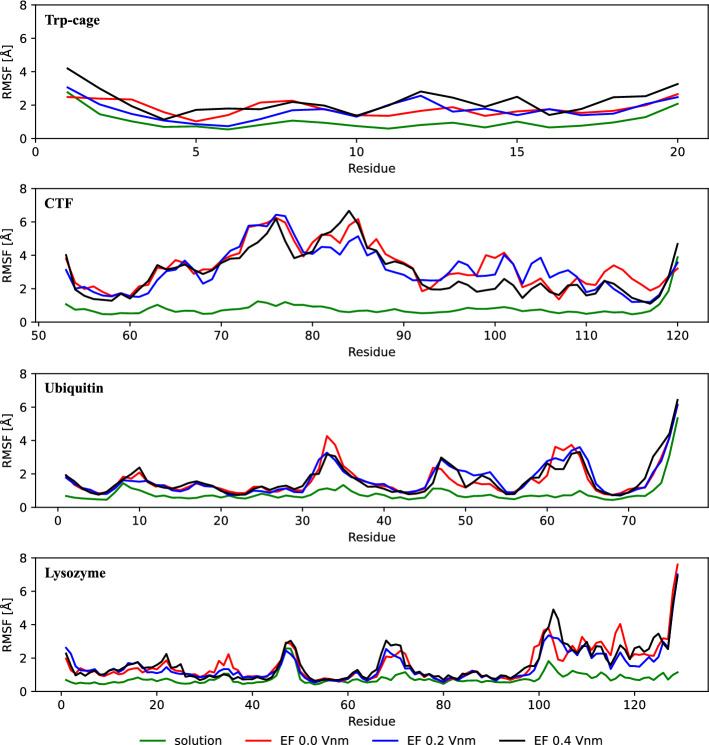
Average fluctuation per residue. RMSF data is presented for structures oriented in a zero, 0.2 and 0.4 V/nm EF strength in red, blue and black, respectively, whilst the solution control data is shown in green. The RMSF calculations display a similar trend of all vacuum-exposed structures for each protein, proposing that the structures exhibit similar fluctuations during rehydration (Color figure online)

To get a more fine-grained view of how the protein structures change upon rehydration, we calculated the per-residue RMSFs from the full trajectories (Fig. 3). RMSFs are time-averages of the squared distance between the instantaneous positions of atoms and a reference structure, which is often the average structure in the trajectory. RMSF thus normally tells about the structural fluctuations around an equilibrium position, where high values correspond to flexible regions. Here however, the structures may change from a vacuum conformation to a solvated one throughout the simulations, and high RMSFs therefore likely indicate parts of the structures where the structural drift is high, whereas low values are more dominated by the fluctuations typically associated with RMSFs.

The RMSFs for the control simulations of all proteins were similar to RMSFs found in literature [45, 49–53]. The RMSFs of the rehydrated proteins were all higher on average than the control solution simulations, which is consistent with their higher RMSDs (Fig. 2). Importantly, the RMSFs were essentially identical regardless of prior field exposure, and we thus instead attribute the difference from the control simulation to prior exposure to vacuum and not to the electric field. Their RMSFs were not always uniformly increased however, instead certain regions were elevated notably more than others, depending on the protein. Trp-cage is an exception, its RMSFs being increased quite uniformly. We attribute this to its small hydrophobic core (Tyr$_3$, Trp$_6$, Leu$_7$, Gly$_{11}$, Pro$_{12}$, Pro$_{18}$, Pro$_{19}$ [54, 55]) reverting back to the center of the protein when reintroduced to a water environment after spending time in the non-hydrophilic vacuum, which requires some rearrangement of all residues in the small protein.

CTF on the other hand showed regions of particularly high RMSF values, and its lowest values were about five residues from the both termini. The RMSFs of the actual termini were high, which is seen for the C-terminus also in the control simulation and in simulations in literature [51]. The first region of relatively low RMSFs up to Ala$_{60}$ forms a $\beta$-strand that is flanked and stabilised by two other strands stretching between Ala$_92$ and Val$_{98}$ and Ala$_{115}$ and Lys$_{120}$ [51]. These $\beta$-strands and the $\alpha$-helix between Lys$_{100}$ to Glu$_{112}$, which make up a small twisted $\beta$-sheet [51], display lower RMSFs than the stretch between Ala$_{61}$ and Pro$_{91}$. This high-RMSF region contains two $\alpha$-helices (Lys$_{65}$–Gly$_{74}$, Leu$_{80}$–Asp$_{87}$), meaning that the RMSF levels are not strictly indicating lack of local structure. The two helices have been shown to have minimal interaction with the water [51], which suggests that their high RMSFs could result from an escape from the protein surface into the protein core, driven by the hydrophobic effect. We note that CTF is the protein in this investigation with the most dramatic charge difference between solution and vacuum, turning from slightly negative to positive (− 2 to + 5). It is known from literature that the isoelectric point, which dictates the net charge at neutral pH, influences the gas-phase stability of electrosprayed proteins [56], where a low value is thought to lead to reduced stability in positive ion mode. In addition to any other features specific to the CTF structure that might make it unstable in the gas phase, its shift from negative to positive is a likely factor behind its high RMSD and RMSF.

Rehydrated ubiquitin had RMSF peaks not seen in the control simulation around Glu$_{34}$, which is the C-terminal end of an $\alpha$-helix, marking the start of a reverse-turn loop leading to the beginning of the $\beta _3$-strand [57, 58]. Another high-RMSF region was seen between Ala$_{46}$ and Glu$_{64}$, containing a minimum corresponding to the 3$_{10}$-helix between Leu$_{56}$ and Tyr$_{59}$ [57] located between two reverse-turns, which are often found near the protein surface [59]. Leu$_{56}$ moreover forms hydrogen bonds with residues in the hydrophobic core, which could provide additional stabilisation. Residues Arg$_{71}$ and upwards make up the C-terminal tail of ubiquitin that extends away from the main protein body, and display high RMSFs in both the rehydrated systems and the control simulations, consistent with high temperature factors in the crystallographic structure [57], suggesting that the high C-terminal RMSFs for rehydrated ubiquitin is at least partly explained by high flexibility and not just structural drift.

The RMSFs from the rehydration of lysozyme were similar to those from the control simulations, apart from a peak around residue 68 and the C-terminal part from residue 100 and onward. A peak around residue Thr$_{47}$ is present for both the rehydration and the control simulations, which corresponds to a $\beta$-hairpin from Thr$_{43}$ to Tyr$_{53}$, which has been reported to exhibit increased mobility, both from experiments and MD simulations [47, 53]. A lack of stabilising hydrogen bonds can explain this peak, and also the peak seen around superficial Pro$_{70}$ in the rehydration simulations, the latter region having high temperature factors in the crystallographic structure [47, 53]. Several low-RMSF regions can be linked to $\alpha$-helices (Cys$_{6}$–His$_{15}$, Leu$_{25}$–Ser$_{36}$, Ile$_{88}$–Val$_{99}$ [47, 53, 59]). The $\alpha$-helix Val$_{109}$–Cys$_{115}$ and the 3$_{10}$-helix Asp$_{119}$ and Ile$_{124}$ however were in the C-terminal region with increased RMSFs. Overall, the RMSFs for lysozyme were comparatively low, which can be attributed to its four disulfide bridges (Cys$_6$–Cys$_{127}$, Cys$_{30}$–Cys$_{115}$, Cys$_{64}$–Cys$_{80}$, Cys$_{76}$–Cys$_{94}$), of which the other proteins have none.

For all proteins, the RMSF calculations indicated that some parts were more mobile during rehydration than the control. The dynamics seemed to occur around similar albeit not identical areas in control and rehydration simulations, suggesting commonalities in the underlying dynamics enabling the structural changes during rehydration.

### Proteins Fully Decompact Upon Rehydration

Vacuum exposure affects protein structures [18, 21, 23], which can ultimately lead to a compaction, manifested as a decrease of their volume, CCS, and surface area [24, 28]. Upon rehydration, back in solution, interactions between the residues and the solvent allow the protein structures to relax and expand, potentially reverting those changes. We first investigated such (de-)compaction by calculating the CCSs for the proteins, which is effectively their average projected areas. The CCS is mostly sensitive to non-occluded surface features [60] and can be inferred from ion-mobility spectrometry for gas-phase proteins [61], allowing for comparison between theoretical values and experiments. We use the CCSs calculated from the rehydration trajectories to detect if major, irreversible structural rearrangements occurred in vacuum, here induced by the EF-orientation or vacuum exposure. Comparing these values to the average CCS from the control simulations reveals similarity or dissimilarity between the two data sets. The SASA and the total volume were also calculated to give additional information about the geometries of Trp-cage, CTF, ubiquitin and lysozyme, displayed with the CCSs in Table 1.

**Table 1:** Size and shape of the proteins in vacuum and solution

Protein	Average collision cross section (CCS) [Å$^2$]							$\Delta$CCS between	
	Solution	EF 0.0 V/nm		EF 0.2 V/nm		EF 0.4 V/nm		Rehydration and	
		Vacuum	Rehydration	Vacuum	Rehydration	Vacuum	Rehydration	Vacuum (%)	Solution (%)
Trp-cage	381 (± 7)	361 (± 4)	386 (± 12)	357 (± 5)	383 (± 12)	357 (± 8)	383 (± 8)	+ 7	+ 1
CTF	819 (± 9)	781 (± 4)	857 (± 28)	791 (± 12)	860 (± 37)	794 (± 10)	874 (± 25)	+ 9	+ 5
Ubiquitin	889 (± 12)	851 (± 14)	929 (± 23)	852 (± 9)	922 (± 21)	841 (± 7)	911 (± 18)	+ 9	+ 4
Lysozyme	1249 (± 11)	1186 (± 10)	1278 (± 21)	1182 (± 12)	1284 (± 18)	1179 (± 12)	1273 (± 20)	+ 8	+ 2

Protein	Average solvent accessible surface area (SASA) [Å$^2$]							$\Delta$SASA between	
	Solution	EF 0.0 V/nm		EF 0.2 V/nm		EF 0.4 V/nm		Rehydration and	
		Vacuum	Rehydration	Vacuum	Rehydration	Vacuum	Rehydration	Vacuum (%)	Solution (%)
Trp-cage	1937 (± 63)	1294 (± 113)	1959 (± 81)	1079 (± 244)	1946 (± 82)	1241 (± 113)	1952 (± 69)	+ 63	+ 1
CTF	4520 (± 110)	3781 (± 179)	4813 (± 226)	3975 (± 282)	4863 (± 248)	3805 (± 160)	4921 (± 227)	+ 26	+ 8
Ubiquitin	4765 (± 109)	3791 (± 25)	5087 (± 165)	4083 (± 143)	5093 (± 177)	3827 (± 177)	4969 (± 162)	+ 30	+ 6
Lysozyme	7052 (± 127)	6035 (± 131)	7335 (± 181)	6013 (± 273)	7367 (± 159)	5954 (± 253)	7347 (± 216)	+ 22	+ 4

Protein	Average volume [Å$^3$]							$\Delta$volume between	
	Solution	EF 0.0 V/nm		EF 0.2 V/nm		EF 0.4 V/nm		Rehydration and	
		Vacuum	Rehydration	Vacuum	Rehydration	Vacuum	Rehydration	Vacuum (%)	Solution (%)
Trp-cage	4743 (± 100)	3171 (± 269)	4750 (± 111)	2624 (± 516)	4750 (± 111)	3070 (± 234)	4753 (± 105)	+ 62	0
CTF	13979 (± 194)	12133 (± 702)	14360 (± 281)	12666 (± 856)	14403 (± 298)	11884 (± 544)	14450 (± 293)	+ 18	+ 3
Ubiquitin	16417 (± 217)	13068 (± 141)	16812 (± 231)	13967 (± 516)	16773 (± 261)	13334 (± 578)	16597 (± 288)	+ 24	+ 2
Lysozyme	26066 (± 298)	23176 (± 784)	26270 (± 357)	23158 (± 1017)	26314 (± 332)	23221 (± 937)	26367 (± 387)	+ 14	+ 1

The average CCS, SASA and volume was calculated for the vacuum structures, and the last 50 ns for the solution and rehydration simulations, respectively. Throughout all obtained data, a reversion of the vacuum compaction can be noticed occurring for all proteins, with the results for the control and rehydration simulations exhibiting similar values

The CCSs for the vacuum structures for all proteins were consistently lower than the solution structures, which is expected from the vacuum compaction. The EF-exposure had no discernible effect on the CCS, neither in vacuum nor after rehydration, differing only by up to about 2%, which is just below the experimental error in ion mobility spectrometry and comparable to the error in the calculations [38]. The rehydrated proteins appear a slight bit larger than the proteins that never left solution, albeit only by a few %. Rehydrated CTF displays the largest difference, 5%, to the control solution simulations, in line with its large RMSDs and RMSFs. Rehydrated ubiquitin also had CCSs that were a bit inflated compared to the control, 4%. These CCSs moreover have larger standard deviations than the other proteins, indicating larger differences between or within replicas, which matches the large RMSD$_f$ and RMSD$_{zf}$ for CTF. Experimental CCSs are 972 Å$^2$ and above for ubiquitin, depending on the charge state [62, 63], which is higher than our calculated CCSs. CCSs depend on the charge state, and the + 7 charge state corresponding to our simulations peaks around 1000 Å$^2$ [63]. Our lower value could be due to the general propensity of the projection approximation algorithm used for the calculations to underestimate CCSs. Applying an empirical correction factor of 1.14 [6], or a power-law calibration to the most rigorous class of CCS algorithms [38], brings our vacuum CCS on par with experiments (970 and 1012 Å$^2$). We note that the net charge of ubiquitin changes from 0 to + 7 when we take it from solution to the gas phase (based on experimental charge state distributions) [15, 21], and the other way around when we rehydrate it. As such, the charge-state shift might put the protein in a inflated state that is not able to fully decompress during the 200 ns of rehydration.

Notwithstanding these slight increases, 200 ns of rehydration appears to be able to revert all or most of the compaction proteins experience in the gas phase, which can be explained by it being largely driven by side-chain interactions on the surface [18, 22], even though cavity collapse can also play a role [24].

SASAs and volumes for the four proteins show a similar trend to that of their CCSs, with lower values for the vacuum structures and a slight increase for the rehydrated structures compared to the controls. Again, values from EF-exposed proteins were not distinct from those from the zero-field counterparts. SASA and volume appear to be more sensitive to structural changes than the CCS, as the relative differences between the values calculated for different conditions for each protein are larger, which we in part can understand based on intrinsic properties of those quantities in a protein context. For convex shapes, the CCS ($\Omega$) and SASA ($A_s$) are related as $\Omega$ = $A_s/4$ under the projection approximation [64, 65], which is approximately correct also under more rigorous theory. Proteins are not perfectly convex however, and the surface can change in ways that do not change the CCS if those changes are relatively small. The larger SASA differences thus indicate surface changes that are not simply compaction, but that can still be explained by rearrangement of surface residues reversing their adaptation to a gas-phase environment [18, 22]. This can be in addition to changes in the backbone structure indicated by the RMSDs and RMSFs. The volume’s dependence on size is $V\approx r^3$, where *r* is the radius, whereas CCS and SASA scales with $r^2$, which is why we see large relative differences there. Comparing the relative volume and SASA changes, we note that the latter are similar or higher than the former while being much larger than the CCS change, which corroborates that there must be surface changes that have a moderate effect on the proteins’ overall shape.

### Contact Maps Suggest Topological Similarities

**Fig. 4:**
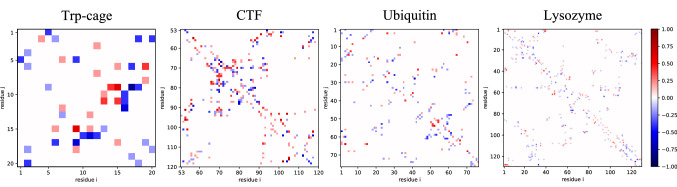
Difference of intramolecular contacts between the vacuum structures. Subtracting contacts present in the 0.0 V/nm EF data set by those present in structures with an EF applied reveals basic differences between the structures. As such, a value of 1 describes contacts that only exist during rehydration, but were not observed during the subtracted data set. Consequently, completely non-existing contacts during rehydration are described by a value of − 1. In the lower triangular matrix, contacts from EF 0.2 V/nm structures were subtracted from EF 0.0 V/nm, whilst the upper matrix shows the subtraction of the EF 0.4 V/nm contacts from those present without an applied EF. Interestingly, some residues seem to be primarily be in contact in the absence of an EF, whilst others are shown to be mostly within close proximity with an EF applied

The RMSDs and RMSFs report on changes to the protein backbones, and the CCS, SASA and volume give information about the overall protein geometry. We have seen previously however that topological features (as defined by residue contacts) might persist also when RMSDs indicate loss of structure, and that they can have an imprint of the pre-vacuum conformation [28]. To see if the orienting EF change the topologies of the structures, we calculated contact maps for the vacuum structures having been exposed to a 0.2 or 0.4 V/nm field, and compared those to the contact maps of the corresponding zero-field vacuum structures, using the structures from Marklund et al. [15]. The contact maps can be seen in Figs. S1–S4, and the maps showing the differences between the oriented and zero-field vacuum structures are shown in Fig. 4 with 0.2 V/nm$-$0.0 V/nm in the lower left triangle, and the 0.4 V/nm$-$0.0 V/nm in the upper right. If the structures are changed by the EF, the difference maps should show the specific changes to the topology.

All oriented proteins exhibited some differences to the zero-field counterparts, indicating that the EF can alter the contact topology. The difference maps are mostly symmetric across the diagonals, but there are also asymmetries, meaning that the 0.2 and 0.4 V/nm systems do have some differences between each other. The vast majority of differences to the zero-field systems are within the $-$0.5 to $-$0.5 range however, meaning that the differences are chiefly not consistent for any of the systems, and it is possible that the differences are actually a manifestation of “natural” variations between the simulations. Variation between replicas for gas-phase simulations of ubiquitin has been reported in literature [48]. Conformations are moreover kinetically trapped in the gas phase [19, 21, 22, 66], making such variations persist throughout MD simulations as well as on experimental timescales. This together with the similarity in magnitude between the map triangles strongly suggests that such conformer variations can at least partially explain the differences seen in Fig. 4. This does not exclude the possibility that the orienting EF can induce changes to the protein topology, which could be what the deep blue or red streaks in the difference maps indicate. For example, for Trp-cage, the contacts with Arg$_{16}$ and residues 10–12 seem to be predominantly present in the oriented structures, and the Asp$_{9}$–Gly$_{15}$ contact is only found in the zero-field structures. CTF has more non-zero elements than ubiquitin and lysozyme, consistent with it having higher RMSDs. Its $\alpha$-helix between Lys$_{65}$ and Gly$_{74}$, and the $\beta$-strand between Ala$_{92}$ and Val$_{98}$, and the contacts they make with the rest of the protein, are more present in the zero-field systems. Interestingly, these regions are not associated with the highest RMSFs, demonstrating that the contact maps reveal structural changes that the other metrics are less sensitive to. Similarly, the contacts involving the $3_{10}$-helix in ubiquitin between Leu$_{56}$ and Tyr$_{59}$ is mostly present in the absence of an EF, but it corresponds to a minimum in the RMSF trace for all rehydration simulations of ubiquitin.

**Fig. 5:**
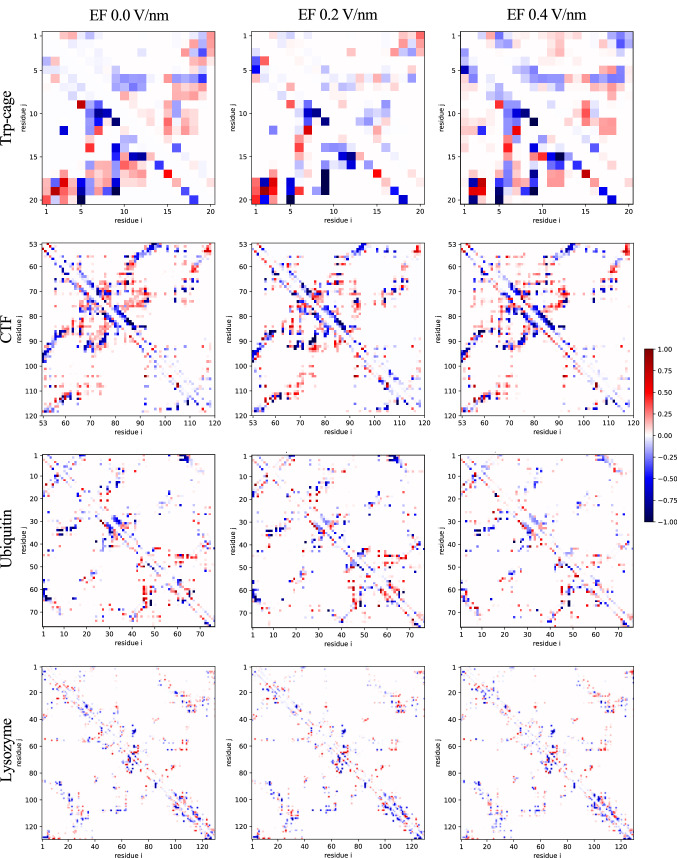
Difference of intramolecular contacts between the data sets over the last 50 ns of simulation. Contacts between residue and residues pairs existing during rehydration were subtracted by contacts existing in vacuum, and during the control solution simulations. In the lower triangular matrix, the difference between rehydration and vacuum contacts are given, in the upper triangular matrix the subtraction of bulk from rehydration contacts

We then turned to the contact maps for the rehydrated proteins (Figs. 5, S1–S4). First, we note that the contact maps for the rehydrated systems are less well-defined than both the vacuum and control solution simulations; both in vacuum and in the control solution, most elements are close to one or zero, indicating stable contacts, whereas the rehydrated systems have features from both the vacuum and control solution simulations, but often at intermediate occupancy. One can see that the rehydrated proteins have lost some vacuum-specific features, but not yet fully recovered all contacts characteristic in solution, and the larger number of intermediate-level elements indicate that the structures might still be changing. Indeed, here, like in Fig. 4, the vast majority of non-zero elements have intermediate values and only a few are close to − 1 or 1. The number of hydrogen bonds to the solvent for Trp-cage and lysozyme was the same after rehydration as in the control simulations. CTF and ubiquitin on the other hand actually had slightly larger numbers of hydrogen bonds with the solvent than the controls did. Vacuum conditions bring about the formation of new intra-protein hydrogen bonds at the surface, and our results indicate a reversal of that process, and that for CTF and ubiquitin the hydrogen bond pattern might still be undergoing rearrangement, with hydrogen bond donors and acceptors being bound to water molecules while their native contacts are yet to materialise. The numbers of hydrogen bonds match with what has been reported in the literature, although interestingly our control simulations for CTF have a lower number than Patriksson et al. [21], whereas rehydrated CTF matched published values almost perfectly. Our results fit with earlier MD studies of protein rehydration, where rapid partial recovery has been seen on short timescales, but not complete recovery for all proteins on timescales similar to our 200-ns simulations [28, 29].

A reoccurring feature that reveals a general difference between the vacuum and rehydration structures is that $\alpha$-helices, seen as streaks offset by three elements from the matrix diagonal, become restored during rehydration. This recovery might not be complete, but the helices are marked notably more faintly in the rehydration difference maps (upper right triangles) than their vacuum counterparts (lower left triangles). Contacts between $\beta$-sheets are also seen as 45$^\circ$-angle streaks parallel or perpendicular to the matrix diagonal (for parallel and anti-parallel $\beta$-sheets, respectively). These are not very prevalent in the difference maps, but are clearly defined in the raw contact maps (Figs. S1–S4). Not all proteins in this study are rich with $\beta$-strands, but ubiquitin have several of them that make up a sheet, and they appear largely preserved or recovered during the process. Contact maps for ubiquitin are available in the literature, and ours match well with the ones for the native structure [46, 67]. CTF in contrast has $\beta$-sheet contacts that appear disrupted in both vacuum and rehydration, but interestingly also some non-native $\beta$-like streaks for residues 80–90 in the 0.0 and 0.4 V/nm (but less for 0.2 V/nm), perhaps partly at the expense of the contacts with residues 53–60. The contacts for the twisted $\beta$-sheet (Glu$_{53}$–Ala$_{60}$, Ala$_{92}$–Val$_{98}$, Ala$_{115}$–Lys$_{120}$) were present, albeit not to 100%, by the end of the rehydration. CTF has so far shown the most clear signs of structural loss, so it is not surprising to see it stand out also in the contact map analysis, but we now see more clearly which parts of the protein rearranges. Trp-cage mostly becomes more similar to the control during rehydration, with no obvious indication that EFs complicates the rehydration. The rehydrated 0.2 V/nm systems in fact are more similar to the control than the zero-field systems are, while the 0.4 V/nm systems are as different as the control or possibly more. Lysozyme have a quite well-preserved structure both in vacuum and rehydration, in line with earlier analyses. Juxtaposing the raw contact maps (Fig. S4) and the rehydration difference maps (Fig. 5), one sees that most features are present, but there are also a number of places where blue and red elements are found next or close to each other in the difference maps, which indicates that some contacts are somewhat perturbed in that contacts have changed to neighbouring residues. Perhaps most notable is a contact between approximately residues 65 and 70 in all rehydrated systems that is absent in the control, which seems to be at the expense of contacts in the control between residue 68 and patches around residue 48 and 60. We recall that the RMSFs for rehydrated lysozyme had a new peak around Pro$_{70}$, which likely reflects the formation of these structural features. The RMSF peak around Thr$_{47}$ is notably not manifested in the difference maps, suggesting similar dynamics for this part of ubiquitin in the rehydrated systems and the control. The lower right corner of the map representing the C-terminal part from Ser$_{100}$ and up show both increased and decreased contacts with neighbouring residues, but few distinct changes in their non-local interactions. Based on the higher RMSFs in this part of the protein in all simulations we assume that the non-zero elements in this part of the difference map is due to structural flexibility that does not average out throughout the course of the simulations.

While there were some contacts that were specific for the rehydrated systems and some that were missing when comparing to the control simulations, it was difficult to discern any EF-specific contacts (or lacking contacts) in the rehydration. And where such contacts can be hinted (for example, between residues 70–80 and 80–90 in CTF), their occupancies were intermediate and could be more “statistical” in nature, originating from variability between simulations. In fact, ubiquitin appears *more* similar to the control after having been exposed to the stronger EF, judging by its paler difference map in Fig. 5, which is more likely to be due to chance than due to the EF. As such, we find that also the contact maps indicate that orienting EFs do not alter the structure significantly, and that any such perturbations are dwarfed by the effect of vacuum exposure in a rehydration simulation.

## Conclusion

The analysis of the MD simulation data presented in this study displays valuable information about EF-exposure of proteins in vacuum and their consequent rehydration. Throughout all data sets and proteins, the results suggest that whilst vacuum-compaction occurs, the applied EFs for dipole-orientation seem to not alter the structures significantly, as all EF-exposed proteins were highly similar to their zero-field counterparts. In fact, the opposite seems to be the case: 200 ns of simulation back in solution was shown to revert the majority of the vacuum-compaction of the proteins, towards similar conformations as provided by the control simulations. However, especially in light of the contact maps, rehydration for 200 ns does not necessarily lead to structures identical to the native state. Regardless, already simulating gas-phase structures in solution on a short time scale of 200 ns generates conformations that are similar to the native state in their contact patterns, suggesting that a longer simulation time in solution could further transfer the structures towards their real standard in solution. Based on our earlier work on the bMS2 dimer, we make the interpretation that the vacuum structures have contact patterns that connect them with their pre-vacuum conformations [28], regardless of whether they have been oriented with strong EFs or not. As such, despite the structures not reverting back completely to their expected conformations in solution during the 200 ns simulations, it is likely that longer simulations or application of sampling techniques would be able to revert the structures. It would be interesting, but beyond the scope of the present study, to explore how large energy barriers stand in the way for complete structural recovery, as that would inform about the recovery time scales and be of practical utility when applying in silico rehydration to vacuum-exposed proteins.

In MS, proteins are exposed to EFs for separation, activation, and for guiding them through the instrument. The field strengths used for such purposes are lower than what is expected to be necessary for orientation, but could in principle have some effect on the structure. As the structural impact is expected to be larger the higher the electric field, our study also show that in MS and other techniques where EFs are applied to manipulate proteins, protein structures are likely to be virtually unaffected by the EFs if the latter are weaker or comparable to field strengths investigated herein. This corroborates recent observations from soft-landing experiments, where native MS is used to select specific proteins and deposit them on surfaces that are later used for electron microscopy [68–71]. The resolution of such experiments have not allowed for atomistic structures, but it is clear that the overall shape of the proteins remain intact under the right condition, even after much longer dehydration times than in our vacuum simulations. Such results also support earlier theoretical investigations by us and others, where electrospraying has not been found to be generally destructive for a protein’s overall structure [19, 21, 22, 29].

Studying the rehydration of proteins is however not only interesting for structural biology, but as well for research areas where the similarities and dissimilarities of proteins in a dry and wet state is of importance. Increasing the shelf life of protein drugs by lyophilization is of significant interest for pharmaceutical companies, where the protein samples are essentially freeze-dried, whilst conserving their biological activity [72]. Investigating this therefore involves investigating the rehydration of dried proteins, as was conducted by Phan-Xuan et al. [27] for example. In their study, the authors examined the stability of lysozyme throughout dehydration and rehydration. The results suggest that the protein underwent structural compaction in the absence of water, but most importantly, during rehydration lysozyme was shown to swell and absorb water. The presented results in this study confirm these observations, where the lysozyme volume increased by 14% upon rehydration. As such, we see great utility of MD simulations for rehydrating vacuum-exposed structures for the investigation of protein-lyophilization, allowing conformational changes to be studied on a level of detail that are inaccessible for other tools and methods.

## Supplementary Information

Below is the link to the electronic supplementary material.

Supplementary file 1 (PDF 1164 kb)
