# Supplementary material for: Rehydration Post-orientation: Investigating Field-Induced Structural Changes via Computational Rehydration
Source: Protein J. 2023 Apr 8;42(3):205–18. doi: 10.1007/s10930-023-10110-y (PMC10264475; doi:10.1007/s10930-023-10110-y)
Supplement: Supplementary file 1 — (PDF 1164 kb) [file 10930_2023_10110_MOESM1_ESM.pdf]

Supplementary Information

Rehydration post-orientation: investigating field-induced structural changes via computational rehydration

Maxim N. Brodmerkel[1], Emiliano De Santis[1,2], Carl Caleman[2,3], and Erik G. Marklund*[1]

[1]Department of Chemistry – BMC, Uppsala University, Box 576, 751 23 Uppsala, Sweden
[2]Department of Physics and Astronomy, Uppsala University, Box 516, 751 20 Uppsala, Sweden
[3]Center for Free-Electron Laser Science, DESY, Notkestrasse 85, 22607 Hamburg, Germany

March 24, 2023

Table S1: **Average hydrogen bonds per data set.** The calculated and averaged number of hydrogen bonds between the proteins and the solvent agree with reported numbers in literature [1].

**Protein**	**solution**	**EF 0.0 V/nm**	**EF 0.2 V/nm**	**EF 0.4 V/nm**
Trp-cage	50 (± 4)	50 (± 4)	50 (± 4)	50 (± 4)
CTF	170 (± 7)	181 (± 9)	180 (± 8)	183 (± 9)
Ubiquitin	181 (± 7)	194 (± 9)	193 (± 9)	189 (± 10)
Lysozyme	282 (± 10)	280 (± 10)	281 (± 11)	279 (± 13)

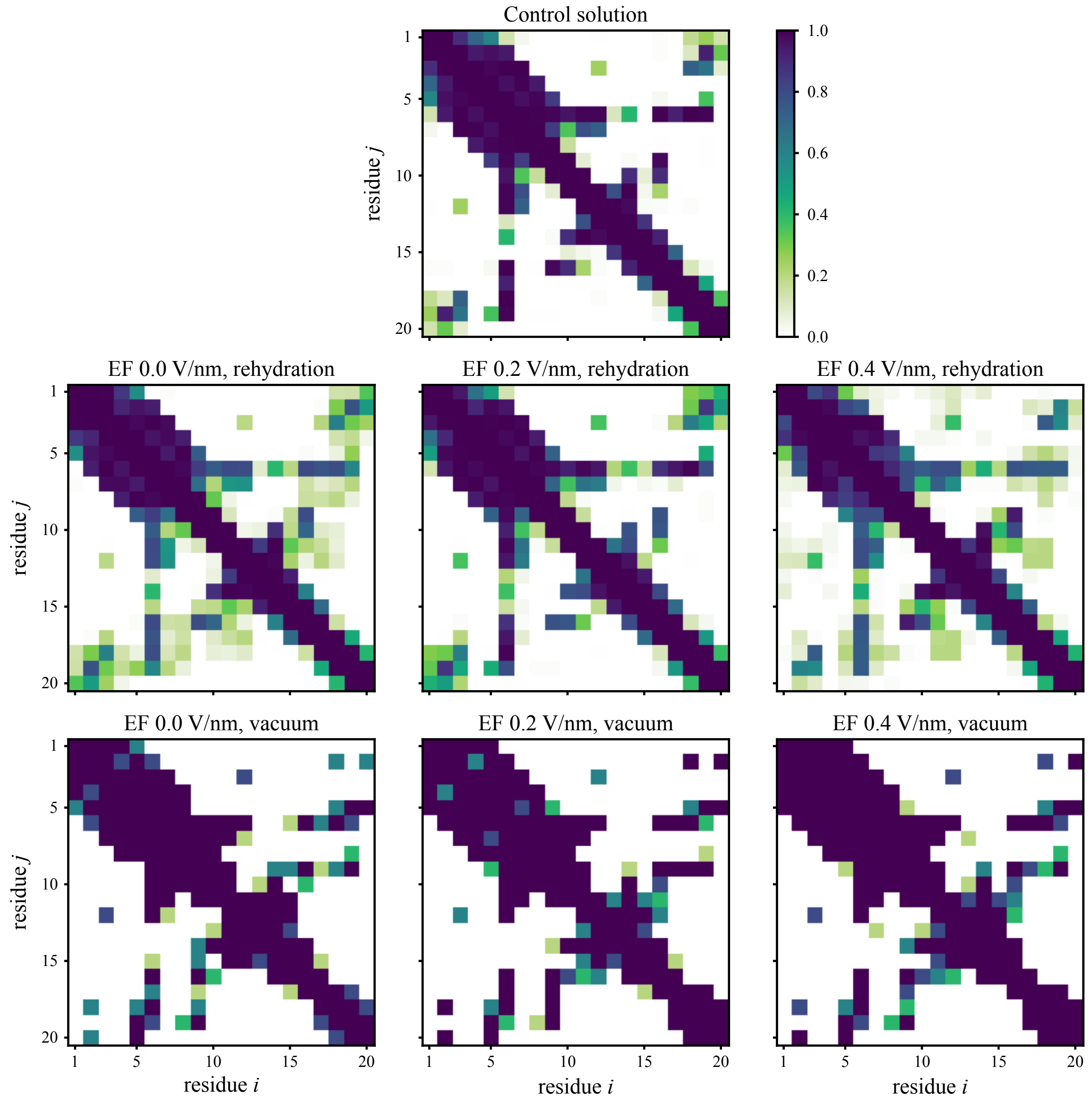

Figure S1: **Contact maps for Trp-cage.**

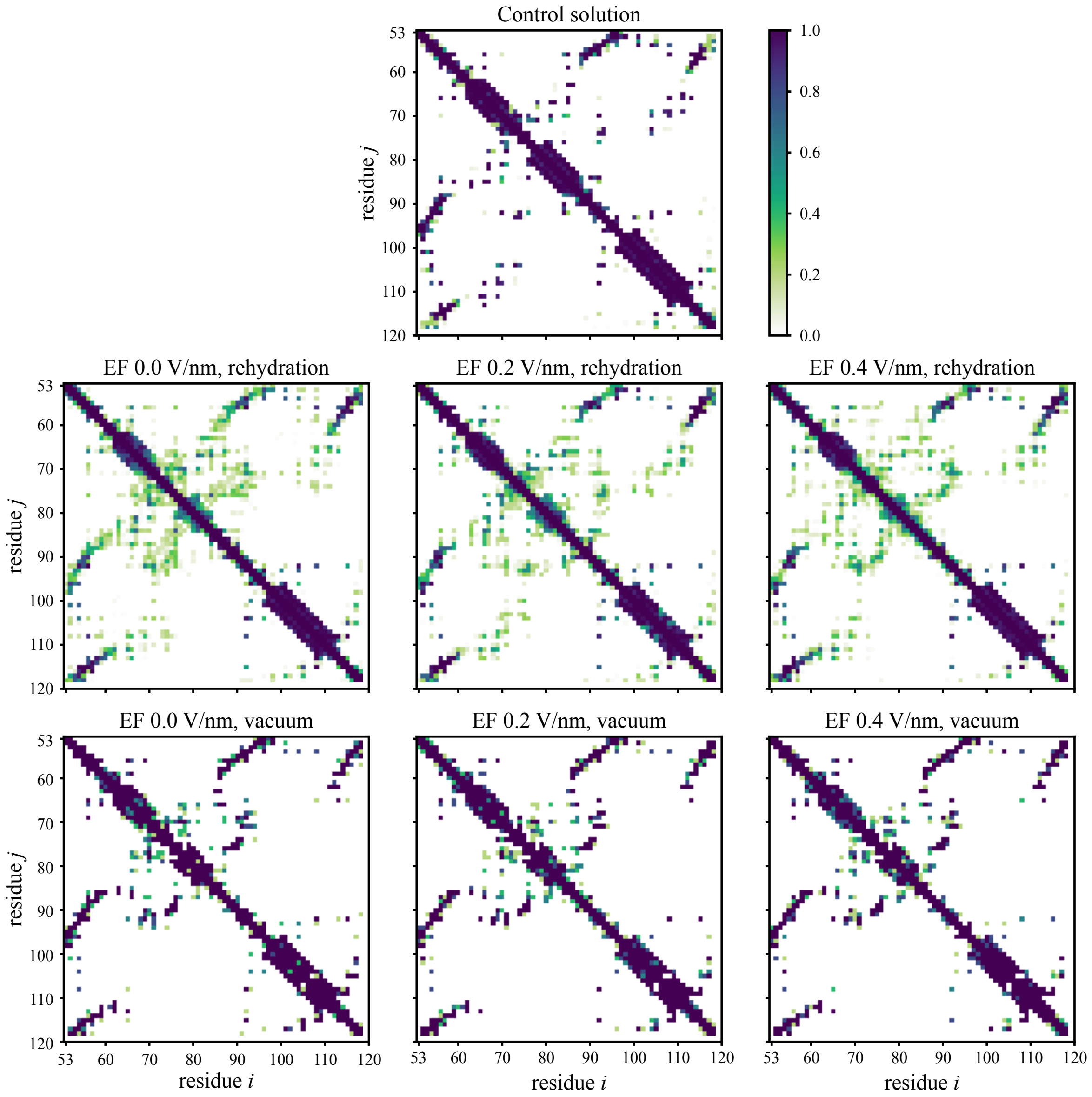

Figure S2: **Contact maps for CTF.**

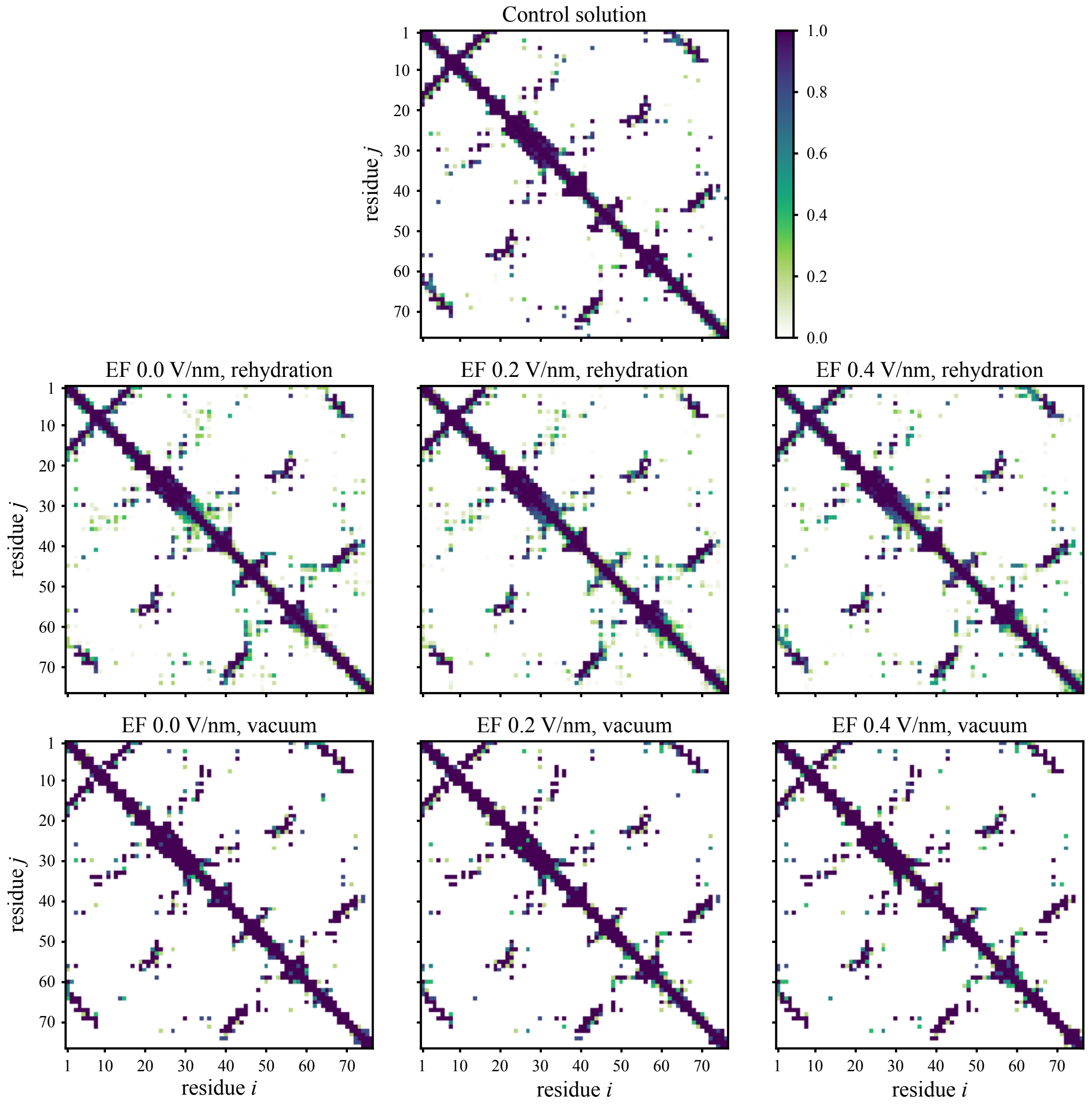

Figure S3: **Contact maps for ubiquitin.**

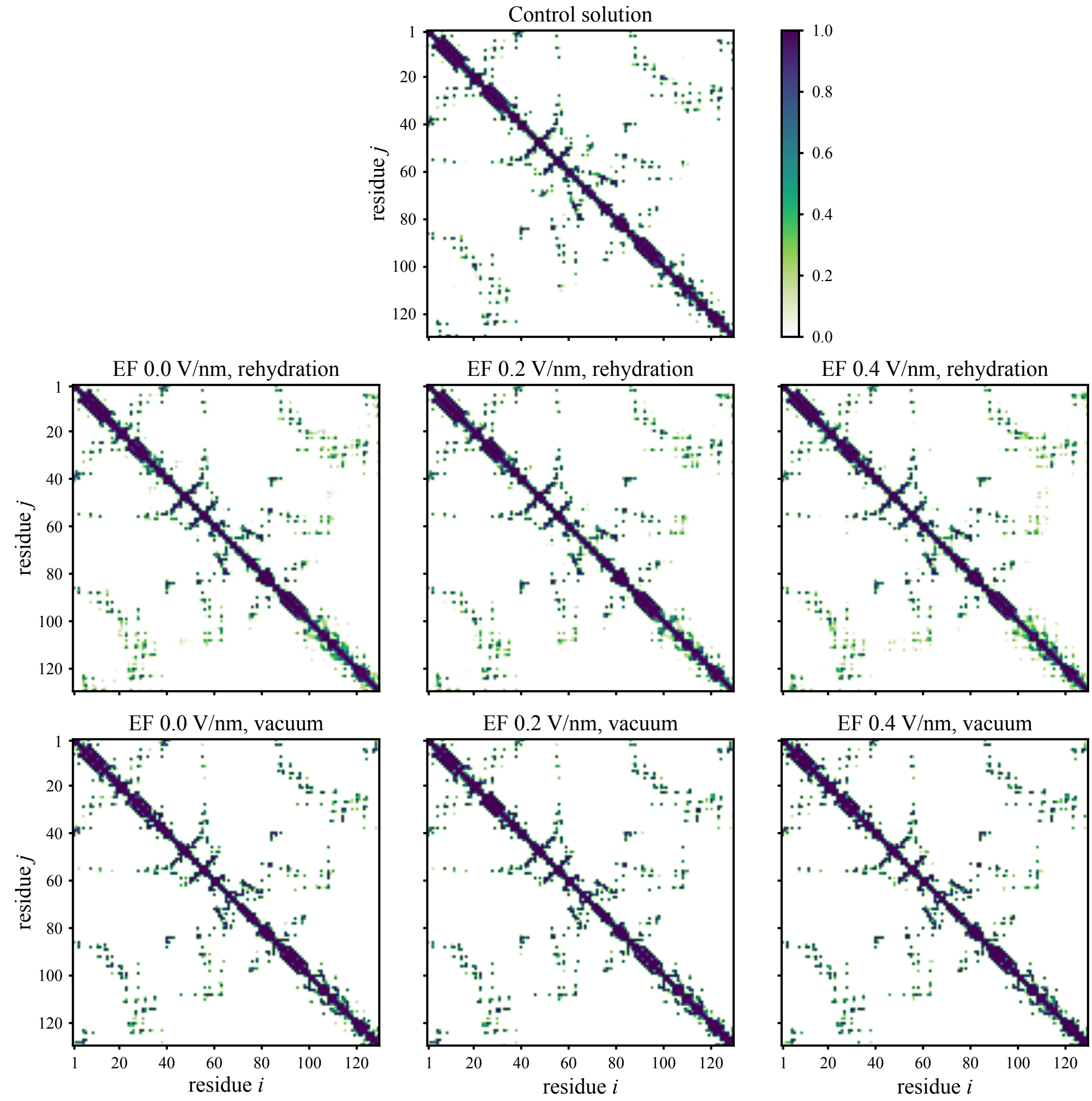

Figure S4: **Contact maps for Lysozyme.**

References

[1] Patriksson, A., Marklund, E. & van der Spoel, D. Protein structures under electrospray conditions. *Biochemistry* **46** (4), 933–945 (2007). https://doi.org/10.1021/bi061182y .
